# Horizontal transfer of aligned Si nanowire arrays and their photoconductive performance

**DOI:** 10.1186/1556-276X-9-661

**Published:** 2014-12-09

**Authors:** Dalin Zhang, Gong Cheng, Jianquan Wang, Chunqian Zhang, Zhi Liu, Yuhua Zuo, Jun Zheng, Chunlai Xue, Chuanbo Li, Buwen Cheng, Qiming Wang

**Affiliations:** 1 State Key Laboratory on Integrated Optoelectronics, Institute of Semiconductors, Chinese Academy of Sciences, QingHua East Road, Haidian District, Beijing, 100083, People’s Republic of China; 2 School of Materials Science & Engineering, Beijing Institute of Technology, South Zhongguancun Street, Haidian District, Beijing, 100081, People’s Republic of China

**Keywords:** Si nanowires, Horizontal transfer, Photoconductive performance

## Abstract

An easy and low-cost method to transfer large-scale horizontally aligned Si nanowires onto a substrate is reported. Si nanowires prepared by metal-assisted chemical etching were assembled and anchored to fabricate multiwire photoconductive devices with standard Si technology. Scanning electron microscopy images showed highly aligned and successfully anchored Si nanowires. Current-voltage tests showed an approximately twofold change in conductivity between the devices in dark and under laser irradiation. Fully reversible light switching ON/OFF response was also achieved with an *I*_ON_/*I*_OFF_ ratio of 230. Dynamic response measurement showed a fast switching feature with response and recovery times of 10.96 and 19.26 ms, respectively.

## Background

Physical properties of one-dimensional (1D) materials are quite different from those of bulk materials because of their distinct features such as high surface-to-volume ratio and quantum confinement effect [1-3]. Therefore, 1D materials, especially 1D semiconductor materials, have drawn much attention during the past decades [4-6]. Silicon nanowires (Si NWs), as a fundamental material in microelectronics, are one of the most attractive 1D semiconductor materials [7-11]. Si NWs have been considered in various potential applications such as in optoelectronics [3,12], electronic devices [13], and energy conversion and storage [14-16]. Bottom-up [17,18] and top-down techniques [11,19,20] are the most common synthesis methods for Si NWs. However, NWs prepared with these techniques are mostly vertically aligned; thus, studying their electron transport features and applications in a variety of devices is a key experimental challenge [21-23].

The main challenge is generally to transfer vertical NWs to a defined position laterally and anchor them with metal electrodes. The most common transfer method mainly involves two steps [3,22]. In the first step, NWs are separated from the growth substrate and dispersed in a volatile solvent. A drop of this solvent is then casted onto the target substrate. In the second step, lithography is used to define the electrode windows, followed by metal deposition and lift-off techniques. However, in this case, NWs are randomly arranged on the substrate. Finding a device with NWs that bridge metal contacts at both ends is time consuming. Moreover, fabricating a device with multiple aligned NWs using the aforementioned method is quite difficult. Several new methods and specific equipment of assembly of nanowires are reported recently [23-27]. Lieber et al*.* developed a nanocombing technique that yields arrays with >98.5% of the NWs aligned to within ±1° of the combing direction [23]. Javey et al*.* used a special print assembled apparatus to print NWs aligned to a receiver substrate [24,25]. Yu et al*.* used a blown-bubble thin film from a solvent containing Si NWs and then stamped a substrate onto the bubble to transfer Si NWs [26]. They transferred uniformly aligned and controlled density NWs onto wafers with a diameter of at least 200 mm. Vertical transfer of Si NW array on glass was also demonstrated by other researchers [27]. In the current study, a simple approach to assemble large-scale and highly aligned Si NW arrays horizontally onto a target substrate surface is proposed. Moreover, multiwire photoconductive devices were fabricated with the assembled NWs. The photoresponse measurements showed a rapid switching property (10.96 and 19.26 ms for the response and recovery times, respectively) and a high I_ON_/I_OFF_ ratio (230). The NW assembly and device fabrication process were easily implemented and cost-effective, i.e., without specific equipment or installation. The proposed method could be a potential candidate for developing large-scale multiwire devices on a flexible substrate.

## Methods

Metal-assisted chemical etching (MACE) method [28] was used to fabricate Si NWs. P-type Si(100) wafers (resistivity *ρ* < 0.01 Ω · cm) were first cleaned before etching. The wafers were rinsed for several times with deionized (DI) water and then dipped into boiling piranha solution [H_2_SO_4_ (95% to 98%) and H_2_O_2_ (30%) at a volume ratio of 3:1] for 3 min to remove metallic and organic residues. The wafers were immersed in diluted hydrofluoric acid solution [DI water:HF (10:1 by volume ratio), 40%] for 30 s to remove native oxide. The as-cleaned samples were etched in MACE solution [0.04 M AgNO_3_ and HF (40%) at a volume ratio of 3:1]. Etching was performed in a water bath at 40°C for 2.5 h with an etching rate of 20 μm/h. All chemicals were of analytical reagent grade and purchased from Sinopharm Chemical Regent Co., Ltd, Beijing, China. In the MACE process, Ag ions were reduced to an Ag dendritic film on a Si wafer, which catalyzed the etching of Si to finally form vertical Si NWs [20]. Ag dendrites formed in the MACE procedure were removed in diluted nitric acid (5 M), rinsed with DI water, and dried naturally.

**Figure 1:**
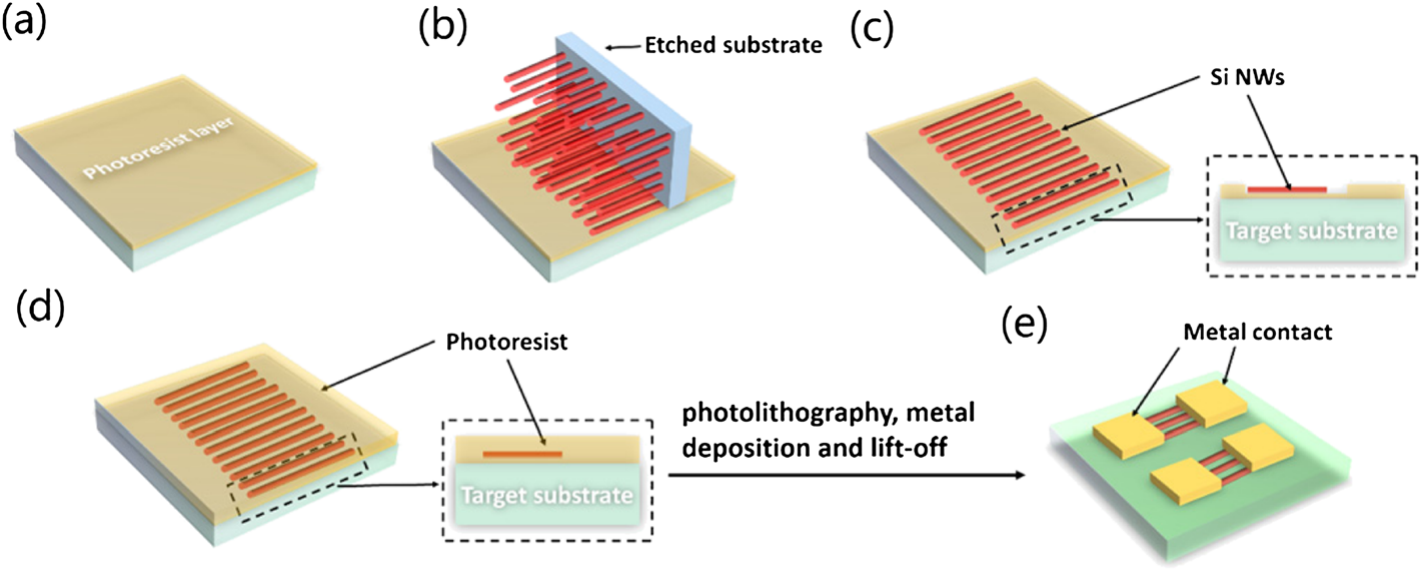
**Schematic diagram of the fabrication of devices with horizontally aligned Si NW arrays. (a)** Photoresist was spin-coated; **(b)** etched substrate was vertically pressed onto the target substrate; **(c)** Si NWs were transferred onto the target substrate; **(d)** the second layer of photoresist was spin-coated; and **(e)** the multiwire devices were successfully fabricated by photolithography and lift-off processes.

To fabricate devices with horizontally transferred Si NW arrays, a SiO_2_/Si substrate was used (SiO_2_ thickness of 200 nm), which was prepared by thermally oxidizing an n-type Si(100) wafer. Figure 1 shows the schematic diagram of multiwire device fabrication. Photoresist with a thickness of 1 μm was first spin-coated onto the target substrate (Figure 1a). To achieve highly aligned NWs, the as-etched Si NW substrate was cleaved into small pieces (1 × 1 cm), which was then pressed vertically onto the photoresist-coated substrate with fresh <110 > cleavage section downward and parallel to the surface of the target substrate (Figure 1b). After removing the etched substrate, a thin layer of Si NW array was stuck into the photoresist (Figure 1c). After heating the sample in an oven at 110°C for 20 min, another 3-μm-thick photoresist was spin-coated (Figure 1d). Simple photolithography (Karl Suss MJB3UV300, SÜSS MicroTec AG, Garching, Germany) was performed to define the anchoring window. The sample was first treated with oxygen plasma (200 W, 1 min, PVA Tepla Plasma System 300, PVA TEPLA, Wattenberg, Germany) to remove residual photoresist and then soaked in diluted HF (10:1 buffered, 5 s) to remove native oxides of NWs, followed by rinsing with DI water and blowing with nitrogen gas to dry. Finally, a 40-nm Cr layer and a 360-nm Au layer were deposited by electron beam evaporation (SKY Tech EB700-I, SKY Tech, Shenyang, China) to anchor the transferred NWs, and the electrodes were formed by a lift-off process. A 1-min rapid thermal annealing at 460°C was performed to form the ohmic contact.

## Results and discussion

**Figure 2:**
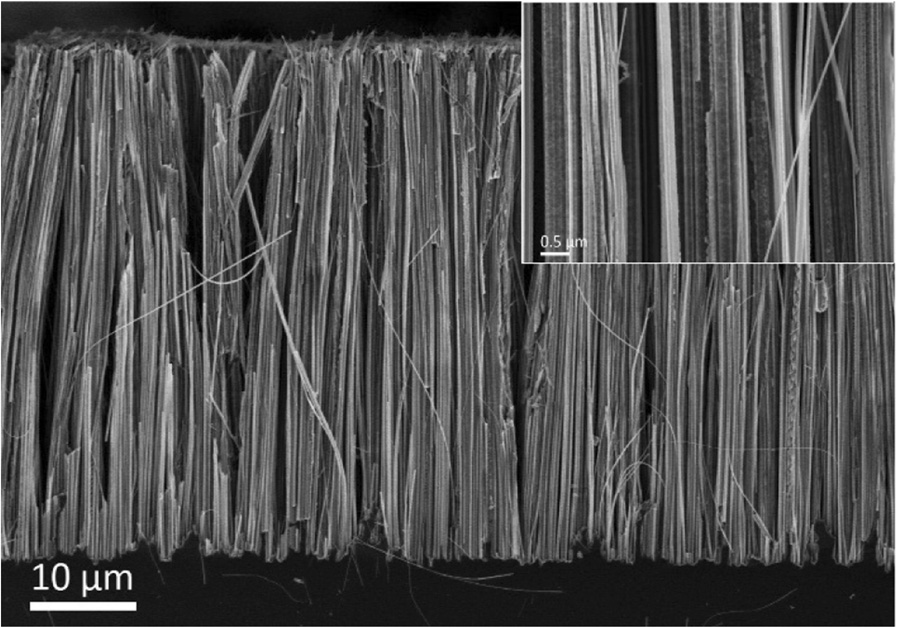
**Cross-sectional SEM images of Si NWs prepared by the MACE method.** The inset shows a high-resolution SEM image. The 50-μm-long NWs are vertically aligned.

**Figure 3:**
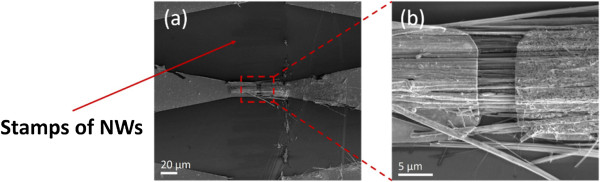
**SEM images and micrograph of multiwire devices. (a)** SEM images of a typical multiwire device; **(b)** high-resolution SEM micrograph of (a). The electrode gaps and electrode widths are 5 and 15 μm, respectively.

The as-prepared Si NWs and horizontally transferred Si NW array devices were characterized by scanning electron microscopy (SEM, FEI Nova NanoSEM 650, FEI, Hillsboro, USA). Dense Si NWs arrays with a diameter of 40 to 400 nm and length of 50 μm were prepared via MACE method (Figure 2). Figure 3 shows the SEM images of devices with horizontally transferred Si NW arrays. The electrode gaps and electrode widths are 5 and 15 μm, respectively. The transferred Si NWs without anchored metal were removed during the lift-off process in acetone (Figure 3a). The stamp marks of the Si NW array left on the substrate are observed, as indicated by the arrow in Figure 3a. However, given the nonuniform pressure distribution along the cleavage edging of the donor substrate during the transfer process, several NWs are still attached to the area outside the devices after lift-off (see Additional file 1). Highly aligned NW arrays are observed (Figure 3b). This process facilitates the designing of the device structure of well-aligned Si NW arrays. Thus, a study on multiwire properties and device application is feasible.

To confirm that aligned Si NW array devices were successfully fabricated, the photoconductive response of the devices was measured using SUSS MicroTec Test Systems (SÜSS MicroTec AG, Garching, Germany) and Agilent B1500A Semiconductor Device Analyzer (Agilent Technologies, Santa Clara, USA). An 808-nm laser diode with a power density of approximately 0.1 W/mm^2^ was used. Current-voltage (*I-V*) characteristics were examined with voltage scanning from -1 to 1 V. Light switching ON/OFF response of the device was measured under a fixed voltage of 1 V. Laser was chopped by an optical chopper (Stanford Research SR540 Optical Chopper, Stanford Research Systems, Sunnyvale, USA) at 3 Hz. All measurements were performed at room temperature.

**Figure 4:**
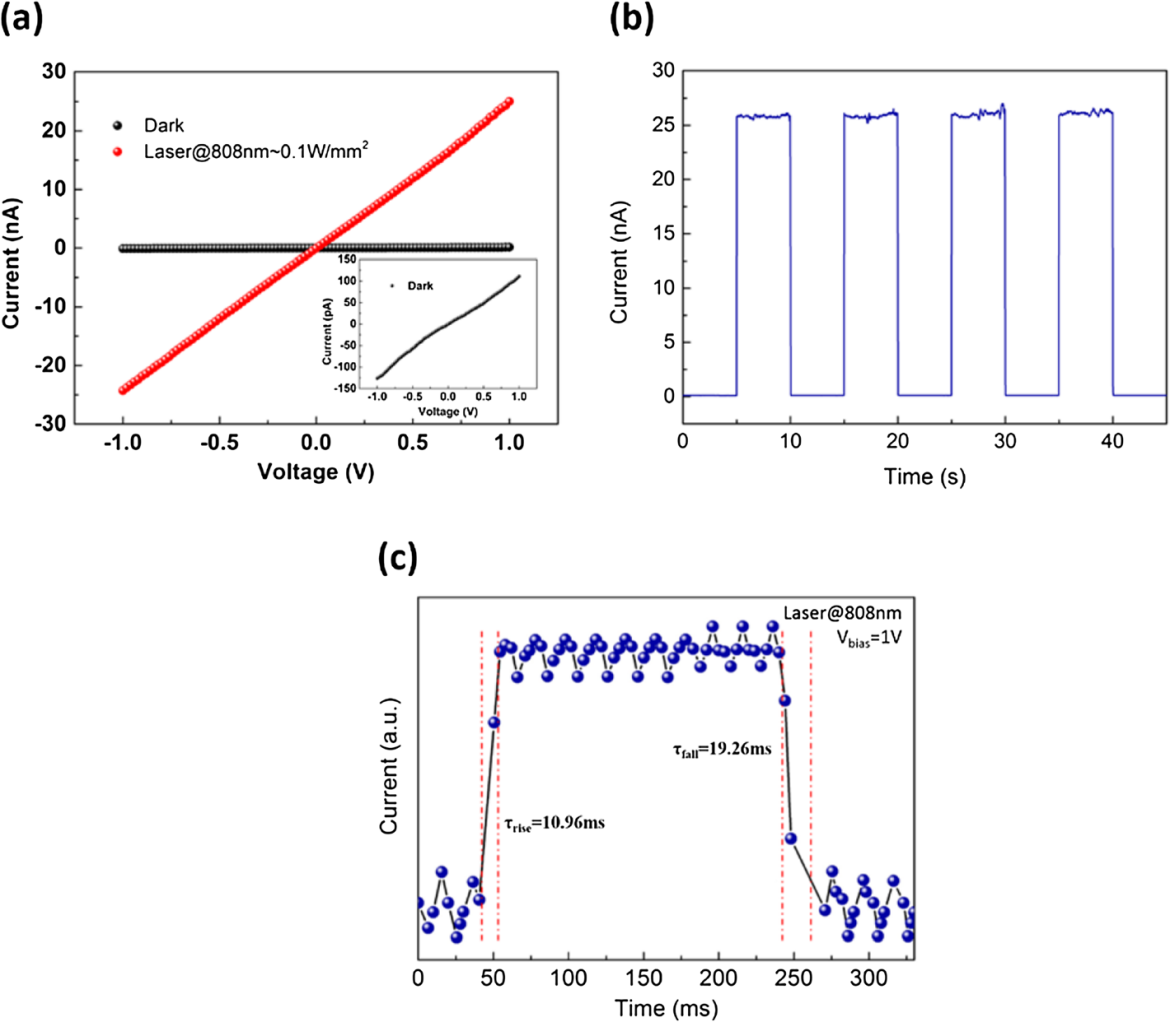
**Photoconductive characteristics of the multiwire device. (a)***I-V* characteristics of the multiwire device in dark (black ball) and under laser irradiation (808-nm wavelength, 0.1 W/mm^2^, red ball); Inset is the *I-V* characteristics of the multiwire device (small scale) in the dark. **(b)** Photoresponse of the multiwire device at a bias voltage of 1 V under laser irradiation (808-nm wavelength, 0.1 W/mm^2^) that were turned ON and OFF. **(c)** Dynamic response performance of the multiwire device at 1-V bias.

The symmetrical linear *I-V* curves of the well-aligned Si NW arrays device from -1 to 1 V both in the dark and with light exposure (Figure 4a) were achieved. Similar *I-V* characteristics were also observed in other devices (see Additional file 1). Using the measured data, the average resistivity of an individual NW was found to be 1.6 × 10 ^5^ Ω•cm, which is much higher than that of the bulk materials. This is mainly attributed to the low dimensionality that reduces the conducting channels and increases the carrier scattering [8,29]. Moreover, a large amount of trap states induced by rough surfaces and interfaces between NWs would reduce the concentration of carriers. In addition, for the multilayer nanowire devices, the electrode metal cannot penetrate into all the layers of the nanowire, and the carrier will overcome a high barrier and introduce high contact resistivity. The resistance decreased remarkably by more than two orders of magnitude by 808-nm laser irradiation compared with that in the dark. This strong conductance enhancement is due to the high surface-to-volume ratio, which allows for more photogenerated carriers. However, the device showed a lower detection efficiency than vertical NW photodetectors. This phenomenon is mainly due to the weak light trap and the thin absorption thickness of the lateral NW structure. Less light is harvested, and fewer amounts of photocarriers are generated [30]. The ON/OFF response of the light switching was measured at 1-V bias (Figure 4b). The device revealed a fully reversible switching characteristic. The conductance remarkably changed with an ON/OFF ratio (I_ON_/I_OFF_) of 230 under laser irradiation and in the dark. This phenomenon suggests that the Si multiwire device is a good candidate for optoelectronic switches, with the illumination and dark corresponding to ‘ON’ and ‘OFF’ states. The response time curve (Figure 4c) shows the time required for the current to increase from 10% to 90% of its steady value, whereas recovery time is defined as the time needed for the current to decrease from 90% to 10% of its steady value [21]. The response and recovery times are 10.96 and 19.26 ms, respectively. Deep trap levels induced by defects and surface states are responsible for prolonged photoresponse time. A passivation process would help promote the response behavior of the NWs. Moreover, the recovery time is relatively slow, which could be attributed to the thermal heating effect of infrared laser [31]. Recombination rate is a function of temperature. However, the temperature variation in the NWs is a slow process considering that their thermal conductivity is greatly reduced because of phonon scattering at the nano-interface [32]. In addition, the trap states of the NW structure also trap the carriers and prevent the recombination of electron and hole [33]. The recovery process fits the following equation [34]:

$$I\left(t\right)\;=\;I_{\text{dark}}\;+\;\Delta Ie^{-\left(t-t_{0}\right)/\tau },$$

where *t*_*0*_ and *t* are the initial and final recovery times, and *τ* is the characteristic time constant, that is, lifetime. *I*_dark_ is the dark current, and Δ*I* is the current amplitude. Accosting to this equation, the lifetime of carriers was extracted from the recovery curve in Figure 4c, and it is determined to be 7.05 ms.

## Conclusions

A simple technique for horizontal transfer of aligned Si NW arrays onto a defined substrate has been demonstrated. Multiwire photoconductive devices were fabricated and tested. The fabricated devices exhibited a twofold change in conductivity between light and dark states. The devices also showed a fully reversible light ON/OFF switching response. High response time (10.96 ms) and recovery time (19.26 ms) were also achieved. The proposed technique provides a facile and cost-effective way to study properties of NWs and planar multiwire device applications.

## Competing interests

The authors declare that they have no competing interests.

## Authors’ contributions

DZ, GC, and CL designed the study and conducted the experiments. DZ, ZL, and CL performed treatment of experimental data and calculations. DZ, CZ, ZL, YZ, JZ, CX, CL, BC, and QW participated in the discussion of the results and initially prepared the manuscript. All authors read and approved the final manuscript.

## Supplementary Material

Additional file 1: Several typical multiwire devices fabricated by horizontal transfer of aligned Si nanowire arrays and their IV characteristics. Click here for file
