# Supplementary material for: Horizontal transfer of aligned Si nanowire arrays and their photoconductive performance
Source: Nanoscale Res Lett. 2014 Dec 9;9(1):661. doi: 10.1186/1556-276X-9-661 (PMC4266530; doi:10.1186/1556-276X-9-661)
Supplement: Additional file 1: — Several typical multiwire devices fabricated by horizontal transfer of aligned Si nanowire arrays and their IV characteristics. [file 1556-276X-9-661-S1.docx]

**Horizontal transfer of aligned Si nanowire arrays and their photoconductive performance**

Dalin Zhang^1^, Gong Cheng^2^, Jianquan Wang^2^, Chunqian Zhang^1^, Zhi Liu^1^, Yuhua Zuo^1^, Zheng Jun^1^, Chunlai Xue^1^, Chuanbo Li^1, a)^, Buwen Cheng^1^ and Qiming Wang^1^

*^1^*State Key Laboratory on Integrated Optoelectronics, Institute of Semiconductors, Chinese Academy of Sciences, Beijing 100083, China

^2^School of Materials Science & Engineering, Beijing Institute of Technology, Beijing 100083, China

Several typical multiwire devices fabricated by Horizontal transfer of aligned Si nanowire arrays are shown in Figure 1s.


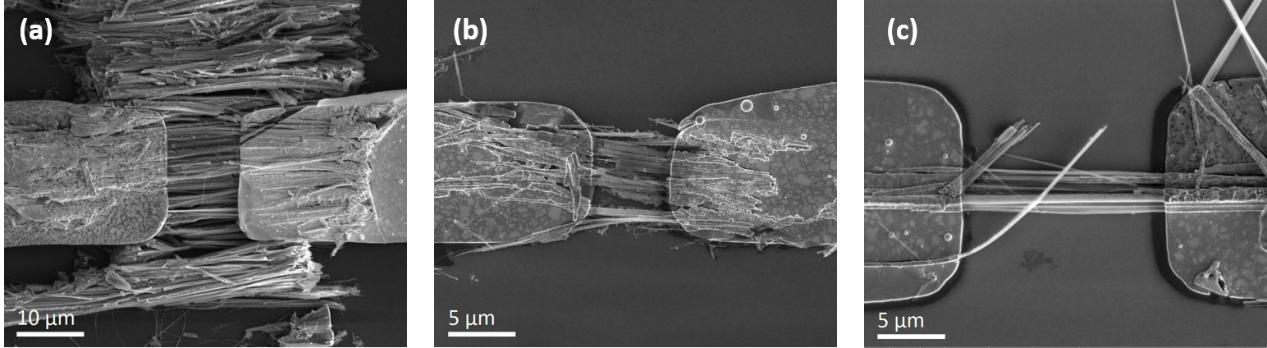


**Figure 1s** SEM images of several typical multiwire devices. The electrodes gaps are 10μm, 5μm and 15μm for (a), (b) and (c). And the electrodes widths are 20μm, 10μm and 15μm for (a), (b) and (c).

All the IV curves of devices exhibit the similar photo-response characteristics as shown in Figure 2s.


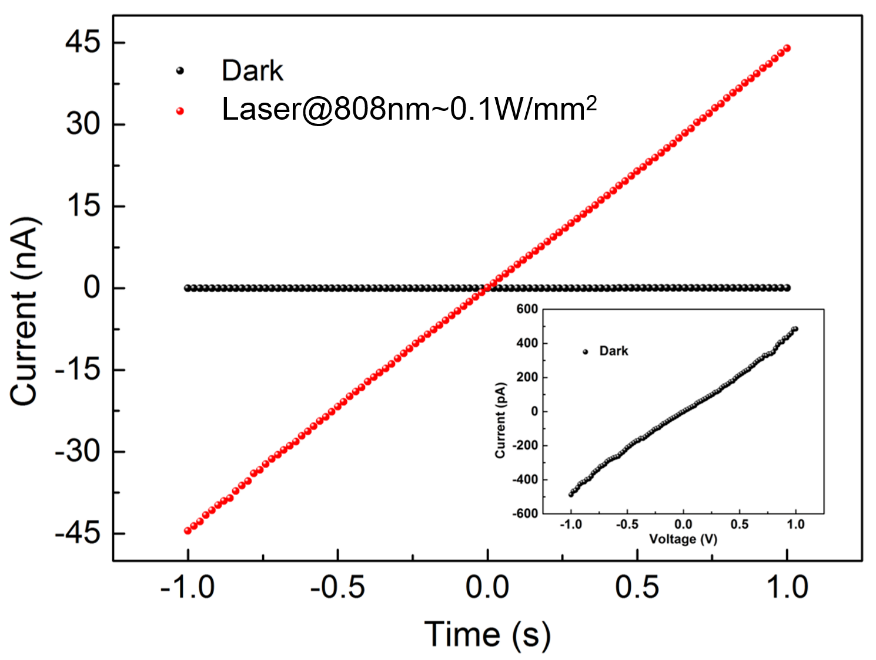


**Figure 2s** *I-V* characteristics of the multiwire device (device (a) in **Fig. 1s** ) in dark (black ball) and under laser illumination (wavelength=808nm ~0.1W/mm^2^, red ball); Insert is *I-V* characteristics of the multiwire device in dark with small scale.
